# Association between media exposure and behavioral problems among preschool children

**DOI:** 10.3389/fpsyg.2023.1080550

**Published:** 2023-07-21

**Authors:** Mohamed A. Zoromba, Doaa Abdelgawad, Sahar Hashem, Heba El-Gazar, Magda Ahmed Abd El Aziz

**Affiliations:** ^1^College of Applied Medical Sciences, Prince Sattam Bin Abdulaziz University, Al-Kharj, Saudi Arabia; ^2^Psychiatric and Mental Health Nursing Department, Mansoura University, Mansoura, Egypt; ^3^Department of Pediatric Nursing, Faculty of Nursing, Mansoura University, Mansoura, Egypt; ^4^Department of Nursing Administration, Faculty of Nursing, Port Said University, Port Said, Egypt

**Keywords:** media exposure, behavioral problems, preschool children, attention deficit hyperactivity disorder, learning disabilities, anxiety

## Abstract

**Background:**

The prevalence of behavioral problems among preschool children is remarkably increasing in the clinical setting.

**Aim:**

The current study aimed to investigate the association between media exposure and behavioral problems among preschool children.

**Methods:**

This survey study recruited 560 children from 10 nurseries selected randomly, located in El-Mansoura, Egypt. Tools included socio-demographic characteristics the Media Exposure Questionnaire and the Conners Comprehensive Behavior Rating Scales (CBRS-48).

**Results:**

The participants were exposed to media for an average of 105.84 min per day and displayed moderate levels in all subscales of CBRS-48. The duration of media exposure was significantly correlated to the hyperactivity index, learning problems, hyperactivity/impulsivity, conduct problem, anxiety, and psychosomatic problems (*r* = 0.372, 0.356, 0.323, 0.306, 0.298, 0.291, and 0.255, respectively).

**Conclusion:**

The duration of media exposure was significantly correlated to the subscales of CBRS-48.

**Implication for nursing practice:**

Preschool children should be engaged in concrete activities and social interactions that may lessen negative media effects such as hyperactivity, learning problems, hyperactivity/impulsivity, conduct problem, anxiety, and psychosomatic problems.

## Introduction

Media has become a prominent feature in the lives of children; media exposure among young children also appears to be on the rise. A recent study shows that media exposure in early childhood has increased by 32% in the last two decades (Goode et al., 2019). Nearly all children (96.6%) use mobile devices, and the majority (92.2%) start using media devices before the age of 1 year. By 2 years of age, many children will have used mobile devices on a daily basis. Typically, 72% of preschool children own at least one screen-based electronic device (Bozzola et al., 2018; Dynia et al., 2021).

The extraordinary childhood exposure to media has evoked interest in its potential impact, where the majority of studies suggest its negative relations (Uzundağ et al., 2022). These correlations include aggressive behavior (Delhove and Greitemeyer, 2021), behavioral problems (Mundy et al., 2017; Monteiro et al., 2021), sleep disorders, anxiety and depression (Scott and Woods, 2019), victimization (Kort-Butler and Habecker, 2018), social isolation (Hammad and Alqarni, 2021), reduced prosocial behavior (Zimmerman and Christakis, 2007), attentional problems, and impairment of cognitive abilities (Christakis et al., 2018).

Specifically, previous studies suggested that the amount of early media exposure is more strongly associated with hyperactivity and inattention problems. Khalil et al. (2022), Mistry et al. (2007), and Pagani et al. (2013) detected long-term associations of exposure to media during early childhood to socioemotional difficulties at school entry and attention problems in the first grade. Moreover, Chassiakos et al. (2016) pointed to the negative effects of media exposure on verbal and memory skills in children aged 6 and 7 years for each additional hour of average exposure to media since 3 years of age.

The significant relationship between the duration of media exposure and later behavioral consequences may result from the decreased interactions between caregivers and children (Barr and Linebarger, 2016). Moreover, exposure to media among preschool children may provide them with cognitively passive activities at a time when key experiences for developing attention and behavioral self-regulation are expected to occur (Radesky et al., 2014). As such, scholars recognize *sensitive periods* during child development, where the specific structures or functions of the human brain are especially vulnerable to particular forms of exposure (Zeanah et al., 2011).

Using data from two time points (child baseline age 2–6 years) and controlling for baseline levels of well-being outcomes, a large cohort study discovered that early exposure to electronic media increased the risk of poorer outcomes in children, but only for some well-being indicators (such as emotional problems), not all of them (e.g., peer problems; Hinkley et al., 2014). Results from a second cohort study that examined the relationship between screen time and behavioral issues at four different ages (ages 3, 5, 7, and 9) revealed that there was a two-way relationship between screen time and internalizing issues (Garfin et al., 2022; Gueron-Sela et al., 2023).

The significance of the study lies in its intention to investigate the increasing prevalence of behavioral problems, such as attention deficit hyperactivity disorders, communication disorders, learning problems, aggression, conduct disorders, and anxiety and psychosomatic problems, among preschool children. As such, the current situation has become remarkable in the clinical setting of child psychiatry (Domoff et al., 2019; Coyne et al., 2021). Thus, determining the risk factors for these emerging problems is considered a valuable role in research. Despite growing concerns about the effects of media exposure on young children, to the best of our knowledge, limited research, specifically from non-western culture and targeted preschool children, has been conducted on the specific relationship between media exposure and behavioral problems in preschool-aged children. To address this gap, a study could be conducted to examine the association between specific types of media exposure (e.g., television, tablet use) and behavioral problems in a large sample of preschool-aged children. Therefore, this study sought to answer the following research questions: How much time are preschool children exposed to media? And What is the relationship between media exposure and behavioral problems in preschool-aged children?

## Subjects and method

### Study design and setting

Current study used the survey study design in accordance with the checklist for reporting of survey studies (CROSS). Ensuing CROSS before and throughout the survey running could support researchers to ensure their survey are satisfactorily reliable, reproducible, and transparent (Sharma et al., 2021). The current study was carried out in nurseries located in El-Mansoura and affiliated with the Ministry of Education in Egypt. According to the Directorate of Education, 33 nurseries are situated throughout the city. The first stage of the study involved a simple random selection of 10 nurseries and during the second stage, students were randomly sampled from each of those nurseries. A total of 70 children were randomly selected using the simple random method from each nursery. Parents were informed about the study formally by letter and informally by verbal messages from the nursery secretaries. A questionnaire and an assessment tool were sent along with the letter, whose subsequent return was considered to be a confirmation of consent after explaining the aim of the study. The response rate of the parents was 80%, during the data collected from September to November 2019. A consultant statistician performed the selection of nurseries and children to ensure randomization.

### Participants

The sample size was calculated using OpenEpi, Version 3, an open-source calculator. According to the Directorate of Education, the hypothesized percentage of the outcome factor in the population (*p*) should be 50% with a precision (*d*) of 5% given a population size (N) of 3,861. Thus, the minimal required sample size (*n*) was 350 with a confidence level of 95%. The sample was increased to compensate for the design effect of multistage sampling. Thus, the final sample obtained was 560 children with consenting parents.

### Tools for data collection

The first part of the questionnaire collected demographic information, including questions about the age, sex, number of siblings, and birth order of the children and posed questions for the parents regarding their age, level of education, occupation, family residence, and family type.

The second part was intended to measure media exposure and was adopted from Ruangdaraganon et al. (2009) and Taylor et al. (2018). The third part posed questions about the knowledge of parents regarding exposure of their children to media, such as the availability of media devices, duration of media exposure, time allotted for the children’s engagement in other activities (e.g., cooking, baking, playing, and bicycling), time for watching TV, content of media, and monitoring of the contents being watched or played by them. We measured media exposure on a *typical day* instead of *the day before* to avoid assessing a day with particularly low or high levels of media exposure compared to the typical exposure of children according to Vandewater and Lee (2009). Furthermore, psychometric properties were assessed to confirm the validity and reliability of the media exposure measure; validity was assessed using the content validity index (CVI), and reliability was assessed using Cronbach’s alpha; (*α*), CVI = 0.76 and *α* = 0.80.

To assess behavioral problems, the Conners Comprehensive Behavior Rating Scales (CBRS-48) was used. The modified Conner’s rating scale consists of 48 items that aim to assess individuals aged 3–17 years. The items are rated using a four-point Likert-type scale ranging from 0 = “Not true at all” to 3 = “Very much true.” Conners CPRS-48 was used to measure six sub-domains, namely, conduct disorders (items 2, 8, 14, 19, 21, 27, 35, and 39), learning problems (items 10, 25, 31, and 37), psychosomatic problems (items 32, 41, 43, and 44), hyperactivity/impulsivity (items 4, 5, 11, and 13), anxiety (items 12, 16, 24, and 47), and item hyperactivity index (items 4, 7, 11, 13, 14, 25, 31, 33, 37, and 38). Because of the aim of the current study was to address children’s behavioral issues, selected items of behavioral issues were used according to the description of the tool subdomains which is drafted with the Arabic version of the tool. The reliability and validity tests of the Arabic version of the Conners CBRS-48, which was developed by Conners, were conducted by Al-Behairy (2011) with positive values, test–retest reliability showed 0.96, 0.89, 0.85, 0.96, 0.90, and 0.98, respectively, for each domain.

A pilot study was conducted on 10 caregivers of children to test the clarity, visibility, and applicability of the tool prior to clinical data collection. The administration of each questionnaire consumed nearly 20 min.

### Ethical considerations

We obtained acceptance to conduct the study from the Faculty of Nursing Ethics Committee, Mansoura University. Moreover, we obtained official approval from the directors of the nurseries and schools. The caregivers of the children were duly informed about the confidentiality of the information collected and that they had the right to withdraw from or refuse to continue the study at any time. Written informed consent was also obtained from the parents regarding data collection and publication.

### Statistical analysis

Data was sorted, coded, organized, categorized, and transferred into especially designed formats. Analysis was performed using SPSS version 24 (IBM Corporation, Chicago, IL, United States). Data normality was first tested using the one-sample Kolmogorov–Smirnov test. The categorical variables were described as numerals and percentage, whereas the continuous variables were presented as mean ± SD (standard deviation). The two groups were compared using Student’s *t*-test. Analysis of variance (ANOVA) was employed to compare the means of more than two groups. Pearson’s correlation was used to determine the correlation between continuous data. Furthermore, the results were considered significant, non-significant, and highly significant when the probabilities of error were <5% (*p* < 0.05), more than 5% (*p* > 0.05), and <0.1% (*p* < 0.001), respectively.

## Results

The results are presented in Table 1, where the mean age of studied children was 3.98 ± 1.73 and more than half (57.1%) were males. In terms of the number of siblings, 41.4% had one sibling, whereas 38.8% were the oldest children according to order of birth. The age of the mothers was 28 years to <39 years (57.7%) and 18 years to <28 years (32%). Half of the mothers (50.2%) achieved secondary education, whereas 31.6% had completed university education. In terms of employment, 51.6% and 45.2% of the mothers were stay-at-home mothers and government employees, respectively. For the fathers, 72% and 20.2% were aged 28–38 years and 39 to <50 years, respectively. In terms of the level of education, 45.4% and 43.6% of the fathers achieved secondary and university education, respectively, and were employed as government employees (52.7%) and in handicraft professions (29.5%). In terms of the family type, the results demonstrated that the children were divided nearly equally between nuclear and extended family types as well as between urban and rural residences. The majority (95.5%) of the families possessed TVs, smart phones, Internet or Wi-Fi services and/or iPads/tablets with less possession of electronic readers and DVD players.

**Table 1:** Personal and demographic characteristics of the children and their parents.

Characteristics		No. = 560	
		No.	%
Children’s age (years) (X̄ ± SD = 3.98 ± 1.73)	Three years or less	152	27.1
	More than 3 years	408	72.9
Children’s gender	Male	320	57.1
	Female	240	42.9
Children’s number of brothers/sisters	No sibling	122	21.8
	One brother/sister	232	41.4
	Two siblings	148	26.4
	Three or more siblings	58	10.4
Children’ birth order	No sibling	102	18.2
	Oldest	217	38.8
	Middle between brothers	96	17.1
	Youngest	145	25.9
Mothers’ age (years) (X̄ ± SD = 30.51 ± 5.97)	18 to <28	179	32
	28 to <39	323	57.7
	39 to <50	50	8.9
	≥50	8	1.4
Mothers’ level of education	Primary/Preparatory	102	18.2
	Secondary	281	50.2
	University	177	31.6
Mothers’ occupation	Governmental work (clerk)	253	45.2
	Freelance (handicraft)	12	2.1
	House wife	289	51.6
	Other	6	1.1
Fathers’ age (years) (X̄ ± SD = 35.6 ± 26.81)	18 to <28	21	3.8
	28 to >39	403	72
	39 to >50	113	20.2
	≥50	23	4.1
Fathers’ level of education	Primary/Preparatory	62	11.1
	Secondary	254	45.4
	University	244	43.6
Fathers’ occupation	Governmental work (clerk)	295	52.7
	Free working (handicraft)	165	29.5
	Not working	32	5.7
	Other	68	12.1
Family type	Nuclear	254	45.4
	Extended	306	54.6
Residence	Urban	265	47.3
	Rural	295	52.7
Availability of entertainment, media materials, and electronic games	1. Television	535	95.5
	2. Play station or videogame player	103	18.4
	3. DVD player	62	11.1
	4. Portable DVD player	116	20.7
	5. Portable play station player	154	27.5
	6. Smart (mobile) phones	491	87.7
	7. iPads/Tablets	293	52.3
	8. Electronic readers	24	4.3
	9. PCs/laptops	408	72.9
	10. Internet or Wi-Fi services	460	82.1

X̄ = mean, SD = Standard deviation.

In Table 2, 25.9%, 25.5%, 20.9%, and 20.5% of the children were exposed to media from 1 h to <2 h, >3 h, <1 h, and from 2 h to <3 h per day, respectively. The mean of the duration of exposure to media was 105.84 min per day. Moreover, 29.6% of the children were engaged in other activities, such as cooking, baking, playing, and bicycling for <1 h. Thus, the mean duration of exposure to other activities was 72.29 min per day. Lastly, 45.5% of the families turned on the TV even if they were not watching.

**Table 2:** Distribution of the parents’ information about their children’s exposure to entertainment, media materials, and electronic games.

Variables	No. = 560	
	No.	%
Duration of children’s exposure to entertainment, media materials, and electronic games		
Not used	40	7.1
<1 h	117	20.9
1 h to <2 h	145	25.9
2 h to <3 h	115	20.5
>3 h	143	25.5
X̄ ± SD	105.84 **±** 79.58	
When the family is at home, how often is the TV turned on even if no one is watching?		
Always	77	13.8
Most of the time	255	45.5
Sometimes	89	15.9
Rarely	114	20.4
Never or absence of TV	25	4.5
Duration of children’s exposure to other activities (cooking, baking, playing, and bicycling)		
Not used	116	20.7
<1 h	166	29.6
1 h to <2 h	116	20.7
2 h to <3 h	72	12.9
>3 h	90	16.1
X̄ ± SD	72.29 **±** 38.46	
Entertainment and media materials frequently watched by the children*		
Cartoon channels	325	58.1
Games on mobile phones and tablets	111	19.8
Educational programs on mobile devices or tablets	13	2.3
Films and series	18	3.2
Is there a TV in the child’s room?		
Yes	139	24.8
No	421	75.2
Does your child own a mobile phone or tablet?		
Yes	127	22.7
No	335	59.8
Shared with siblings	98	17.5
Are you monitoring what your child watches or plays?		
Always	145	25.9
Most of time	207	37
Maybe	193	34.5
Never	15	2.7

* More than one response.

X̄ = Mean, SD = Standard deviation.

Regarding the type of media that the children were exposed to, 58.1% and 19.8% were exposed to cartoon channels followed by mobile and tablet games, whereas 24.8% of the children had TVs in their rooms. Moreover, 22.7% owned mobile devices or tablets, whereas 17.5% shared a mobile device or tablet with their siblings. Lastly, 62.9% of the parents monitored what their children were watching or playing.

**Figure 1:**
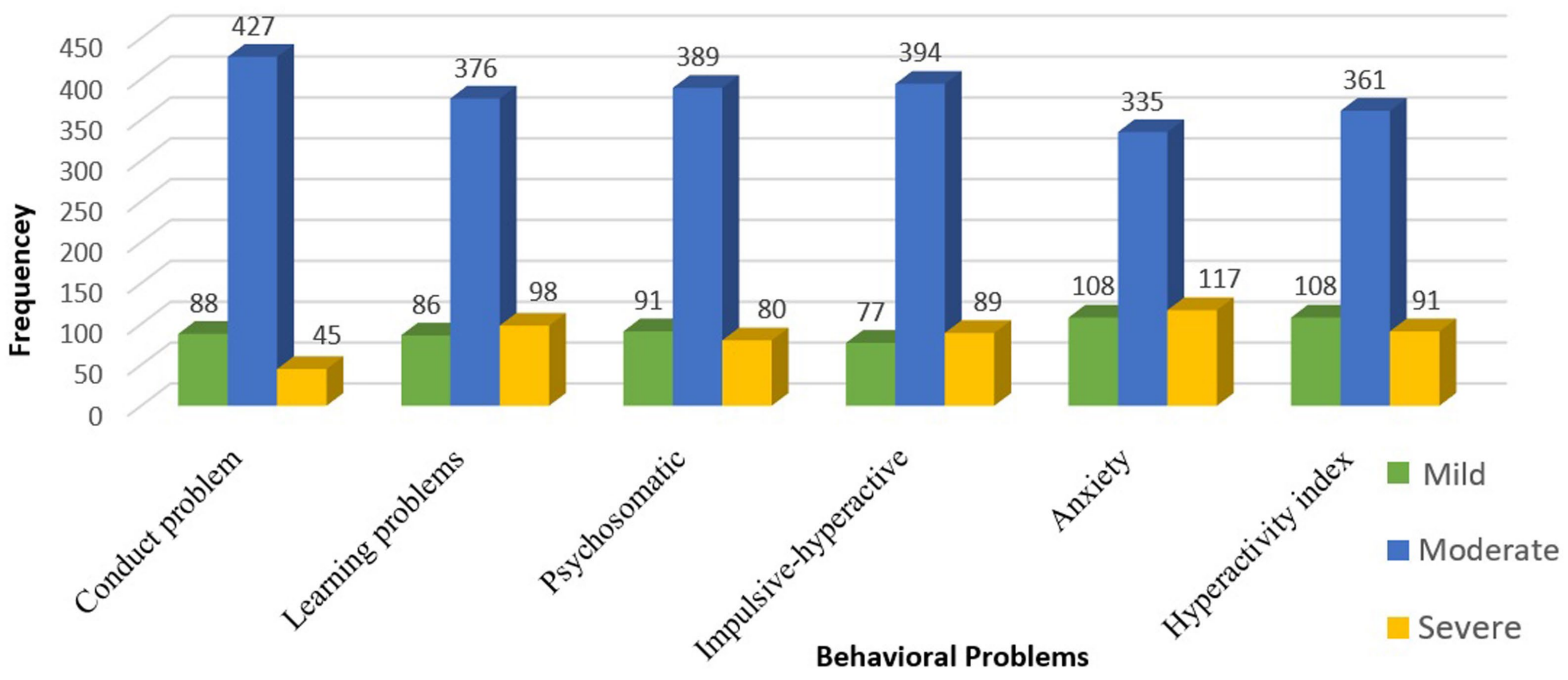
Levels of behavioral problems among the children (*n* = 560).

Figure 1 illustrates that the preschool children displayed moderate levels in all subscales of Conners CBRS-48. In particular, 20.9% of the children exhibited severe levels of anxiety, whereas 17.5% showed severe levels of learning problems. Lastly, 19.3% of the children demonstrated mild levels of anxiety and hyperactivity.

Table 3 illustrates that the duration of media exposure was significantly and positively correlated to Conners CBRS-48, the hyperactivity index, learning problems, hyperactivity/impulsivity, conduct problem, anxiety, and psychosomatic problems (*r* = 0.372, 0.356, 0.323, 0.306, 0.298, 0.291, and 0.255, respectively). No significant correlation was observed between the duration of media exposure and exposure to other activities where parents engaged with children.

**Table 3:** Duration of media exposure in correlation with exposure to other activities spent with children and the Conners Comprehensive Behavior Rating Scales (CBRS-48; *n* = 560).

Media exposure	Test of Sig.	
	*r*	Value of *p*
Duration of other activities spent with the children	0.072	0.089
Conduct problem (8 items)	0.298	<0.001
Learning problems (4 items)	0.323	<0.001
Psychosomatic (4 items)	0.255	<0.001
Hyperactivity/Impulsivity (4 items)	0.306	<0.001
Anxiety (4 items)	0.291	<0.001
Hyperactivity index (10 items)	0.356	<0.001
Total (CBRS-48; 48 items)	0.372	<0.001

*r* = Pearson’s correlation; *p* = significant at <5%.

Table 4 clarifies multivariate analysis of variance (MANOVA) addressing differences across studied variables, accordingly, there is a significant effect of media exposure as independent variable on behavioral problems as dependent variables among studied preschool children, F (Roy’s Largest Root) = 7.466 with significant level *p* < 0.001.

**Table 4:** The one-way multivariate analysis of variance (one-way MANOVA) addressing differences across studied variables (*n* = 560).

Media exposure	Conduct problem		Learning problems		Psychosomatic		Hyperactivity/Impulsivity		Anxiety		Hyperactivity index		Total CBRS -48	
	*M*	SD	*M*	SD	*M*	SD	*M*	SD	*M*	SD	*M*	SD	*M*	SD
Not used	8.85	4.59	4.53	2.88	3.35	2.50	5.93	2.64	4.93	2.67	13.40	5.79	54.48	26.29
<1 h	9.33	4.51	4.18	2.59	3.17	2.30	6.63	2.65	5.01	2.41	14.08	6.40	52.96	21.04
1 h to <2 h	8.47	4.08	4.14	2.52	3.68	2.52	6.03	2.79	4.70	2.63	13.12	5.97	52.04	21.51
From 2 h to <3 h	9.38	3.90	4.03	2.40	3.59	2.82	7.39	2.65	4.83	2.55	14.06	5.20	55.09	19.07
3 h and more	9.15	4.31	4.86	2.74	3.76	2.79	7.00	2.62	4.99	2.58	14.98	6.14	56.17	23.35

*F* (Roy’s Largest Root) = 7.466, Sig < 0.001, and partial Eta Squared = 0.086, *M*, Mean; SD, standard deviation; CBRS, Conners Comprehensive Behavior Rating Scales.

## Discussion

The World Health Organization (WHO) recommends limiting preschoolers’ average screen time to 1 hour a day by restricting their access to TVs and other media devices (WHO, 2019). Consistent with those of previous studies, our results suggest that in the majority of homes, these recommendations are far from reality. In fact, several media types are nearly a constant presence in the lives of all children.

The brain of preschool children develops rapidly and is characterized by relative plasticity in response to the environment (Gilmore et al., 2018). The media, nowadays, is considered as one of the elements that influences the children’s cognition (Blumberg et al., 2019). Accordingly, the current study demonstrated that less than one-quarter, more than one-quarter, and more than one-fifth of the preschool children were daily exposed to media from 1 h to <2 h, >3 h, and from 2 h to <3 h, respectively. The mean duration of exposure to media was 105.84 min per day.

The literature provides varied results. For example, Tomopoulos et al. (2007) reported that preschool children were exposed to media for 128.9 min/day, whereas Munzer et al. (2018) stated that the mean screen media exposure was 156 min/day. This result is in agreement with that of Kovačič and Rek (2016), who found that preschool children were exposed to media for 159.85 min/day. Moreover, the mothers in the study of Waters et al. (2016) declared an average of 177 min of electronic media use on weekdays. Moreover, Conners-Burrow et al. (2011) observed that children watched TV for198 min/day.

The study findings showed that the majority of the children were aged more than 5 years and more than half were males. In terms of the number of siblings, 41.4% had one sibling, whereas 38.8% were the oldest children according to order of birth. Half of the mothers achieved secondary education, whereas 31.6% had completed university education. The vast majority of the families possessed TVs, smartphones, Internet or Wi-Fi services and/or iPads/tablets with less possession of electronic readers and DVD players. This suggests that the sample consisted mostly of older children and that there was a relatively equal gender distribution. Also, the studied sample consisted of a mix of children with different family structures and birth order positions. Results suggested that the majority of the mothers were between the ages of 28 and 39, with a smaller proportion being younger, they were with a mix of educational backgrounds, additionally, the vast majority of the families had access to modern technology, which may have implications for their children’s media exposure and behavioral problems.

Variance regarding the duration of media exposure is related to many factors, such as birth order, residence, mothers’ age, her level of education and occupation, culture, content, screen availability, and family economic status (Eales et al., 2021). Studies on different cultures reported that the level of exposure of preschool children to media is higher than that recommended by the American Academy of Pediatrics (2001), WHO (2019) and Domoff et al. (2020). Evidently, children are spending considerable amounts of times in front of the screen in a passive manner instead of being physically active. Thus, media devices have become an influential factor in the lives of children (Robinson et al., 2017; Domoff et al., 2020).

The current study revealed that the duration of media exposure is significantly and positively correlated to Conners CBRS-48, the hyperactivity index, learning problems, hyperactivity/impulsivity, conduct problems, anxiety, and psychosomatic problems. Congruently, a meta-analysis conducted by Nikkelen et al. (2014) noted a significant relationship between media use and behaviors related to attention deficit hyperactivity disorder.

Radesky et al. (2014) found that mild increases in media exposure are associated with problems related to early childhood self-regulation. Moreover, sustained TV exposure is a risk factor for behavioral problems (Mistry et al., 2007). Specifically, Xie et al. (2020) observed that preschool children with a screen time of more than 60 min tend to display more behavioral problems than those with a screen time of <60 min.

Along these arguments, Parkes et al. (2013) reported that watching TV for 3 h or more among 5-year old children predicted conduct problems by the time they reach 7 years compared with watching it <1 h. In addition, other studies addressed the relationship between media content and behavioral problems. For example, Conners-Burrow et al. (2011) confirmed that viewing videos/movies that are rated as inappropriate for young children was associated with increased hyperactivity and aggression scores and low levels in social skills.

In contrast, Conners-Burrow et al. (2011) reported that the duration of media viewing was non-significantly associated with hyperactivity and aggression. Similarly, Zimmerman and Christakis (2007) stated that viewing any content at ages 4–5 years was not associated with attentional problems. Parkes et al. (2013) reported that playing electronic games was not associated with conduct problems, moreover, there were no associations between the type of media and emotional symptoms, hyperactivity/inattention, peer relationship problems, or prosocial behavior.

The duration and content of media exposure among preschool children widely varied according to culture. Moreover, content considered appropriate for adults may be perceived by children negatively (Guellai et al., 2022). In general, preschool children may imitate what they are exposed to. Simple animations may influence children’s cognition, which is largely undeveloped, causing them to think and act impulsively or aggressively, similar to the characters they see. Moreover, complications may arise when children spend long periods of time in front of screens or are exposed to content that is inappropriate for their age. Finally, reducing the duration of media exposure can provide children with ample opportunities for interaction with peers, parents, and the community (Sanders et al., 2018).

In conclusion, the current study revealed that preschool children are generally exposed to media for an average of 105.84 min/day. Moreover, the duration of media exposure is significantly and positively correlated to Conners CBRS-48, hyperactivity index, learning problems, hyperactivity/impulsivity, conduct problems, anxiety, and psychosomatic problems. Therefore, the study highlights the need to engage preschool children in concrete activities, familial and social interactions that may lessen negative media effects such as hyperactivity, learning problems, hyperactivity/impulsivity, conduct problem, anxiety, and psychosomatic problems. As such, future studies should examine other data in detail to explore the risks of various forms of screen time and media content, including exposure to screen violence. Lastly, longitudinal studies are required to examine the long-term effect of media exposure on preschool children particularly after pandemic of COVID-19. Studies may assess the effect of pandemic on exposing preschool children to media besides its consequences.

What is already known on this topic:

- Increased exposure to media has been linked with behavioral problems, although previous findings are inconsistent.
- The majority of studies have focused on watching TV only and overlooked other types of media that have currently emerged and invaded children’s lives.
- Few studies have examined media exposure within specific age groups, such as preschool children.

What this study adds:

- The preschool children were exposed to media for an average of 105.84 min/day.
- The preschool children displayed moderate levels in all subscales of Conners CBRS-48.
- The duration of media exposure is significantly and positively correlated to Conners CBRS-48, hyperactivity index, learning problems, hyperactivity/impulsivity, conduct problem, and anxiety and psychosomatic problems.

## Data availability statement

The original contributions presented in the study are included in the article/supplementary material, further inquiries can be directed to the corresponding author.

## Ethics statement

The studies involving human participants were reviewed and approved by Research Ethics Committee of the Faculty of Nursing, Mansoura University. Written informed consent to participate in this study was provided by the participants’ legal guardian/next of kin.

## Author contributions

MZ and MA planned the study. DA and MZ analyzed the literature. MZ, SH, and DA were major contributors in writing the results section. MZ, HE-G, and MA were major contributors in writing the discussion section. All authors contributed to the article and approved the submitted version.

## Conflict of interest

The authors declare that the research was conducted in the absence of any commercial or financial relationships that could be construed as a potential conflict of interest.

## Publisher’s note

All claims expressed in this article are solely those of the authors and do not necessarily represent those of their affiliated organizations, or those of the publisher, the editors and the reviewers. Any product that may be evaluated in this article, or claim that may be made by its manufacturer, is not guaranteed or endorsed by the publisher.
